# Hannibal’s Ophthalmia—A New Answer to An Ancient Question

**DOI:** 10.3201/eid2701.191696

**Published:** 2021-01

**Authors:** Justin T. Denholm, Patrick N. Hunt

**Affiliations:** Royal Melbourne Hospital, Parkville, Victoria, Australia (J.T. Denholm);; University of Melbourne, Parkville (J.T. Denholm);; Stanford University, Palo Alto, California USA (P.N. Hunt)

**Keywords:** Hannibal’s ophthalmia, ophthalmia, Hannibal, Pseudomonas aeruginosa, Acanthamoeba spp., bacteria, eyes, inflammatory eye disease, keratinitis, elephant-associated zoonoses, pachyderm-associated zoonoses, zoonoses

## Abstract

In the spring of 217 bce, shortly after Hannibal’s famous elephantborne crossing of the Alps, the general was afflicted by an acute, painful eye condition that has never been adequately explained and that led to permanent unilateral loss of vision in 1 eye. In modern times, scant attention has been given to understanding this condition. We review the historical and geographic evidence and consider possible infective explanations for Hannibal's condition, including elephant-associated zoonoses. Ultimately, we suggest that a keratitis from waterborne organisms, such as *Pseudomonas* spp. or *Acanthamoeba* spp., might provide the best answer to this ancient enigma.

In the spring of 217 bce, shortly after Hannibal’s famous elephant-borne crossing of the Alps, the general was afflicted by an acute, painful eye condition that has never been adequately explained. The Roman historian Polybius’ account in Greek of Hannibal’s affliction is brief, stating, “Hannibal himself on the sole remaining elephant got across with much difficulty and suffering, being in great pain from a severe attack of ophthalmia (Greek ’οφθαλμια), which finally led to the loss of one eye as he had no time to stop and apply any treatment to it, the circumstances rendering that impossible” (*1*). Earlier passages set the scene of a grueling 3-day march through the marshes of the Arno River, as Hannibal once again attempted to take his Roman opponents by surprise by arriving just west of Arezzo (ancient Arretium; “the back way”). Although his bold movement was militarily successful, the illness he acquired led to the permanent loss of vision in 1 eye. This affliction became a defining characteristic of Hannibal’s portrayal in history, as seen how his visual loss is referenced as influencing his actions on campaign, and in later artistic depictions of the general (*2*).

## Historical Arno Valley Swamps

Hannibal’s marsh crossing most likely took place in the middle Arno River Valley in upper Tuscany in northern central Italy, just east of modern Florence. Late Quaternary sedimentology of the mountain-rimmed Arno River watershed has documented long-term drainage problems with marshlands both coastal and inland, and peat deposits in the Arno Valley also attest to old bog contexts (*3*,*4*). Although land reclamation in the late 1800s finally reduced the perennial flooding in the Florentine floodplain region, the middle Arno valley was once a series of ancient lakes with a pervasive semipermanent swamp area called le padule and many small bogs still exist (*5*). Occasional flooding of the region still occurs, with the most recent catastrophic instances in 1966 and 1987, which damaged or threatened many notable works of art.

## Ophthalmia in the Ancient World

A diverse range of eye diseases were recognized and described in antiquity (*6*). The ocular process that caused Hannibal’s blindness has been generally referred to as ophthalmia, following the term used by Polybius. Elsewhere in the ancient world, this term seems mainly to describe inflammatory eye disease, whether acute or chronic, without evidence that a particular disease process is implied. Ophthalmia was described by Hippocrates, including treatment regimens, and by the time of the Roman Empire, specialist eye doctors (medicus ocularius) were recognized (*7*). Hippocrates also tells us that ophthalmia was more common in the spring, at the time Hannibal went through the marshes, and that there is an association with stagnant and muddy conditions (*7*,*8*). Herodotus reported that 2 Spartans were afflicted with ophthalmia before the battle of Thermopylae, also in the context of nearby marshlands (*9*). Neither, however, provides further insight into the likely cause of this disease.

In instances in which further clarification has been offered, later historians have sometimes labeled Hannibal’s ophthalmia as a form of conjunctivitis, perhaps contracted, as Polybius noted, after “long periods without sleep weakened [Hannibal’s] resistance” (*10*). However, there are various reasons why this diagnosis might be questioned. Viral conjunctivitis most commonly progresses to become bilateral, and although bacterial infections might remain unilateral, it is uncommon for pain to be a severe manifestation of this disease (*11*). Regardless of the etiologic pathogen, conjunctivitis is more common in childhood and normally resolves without sequelae. Hannibal’s illness, then, as an adult manifestation with severe unilateral pain and ultimate permanent loss of vision, is not strongly suggestive of a simple conjunctivitis.

## Diagnosis

If history has been hasty in arriving at such a conclusion, does the contemporary historical record permit a more likely diagnosis to be reached? The list of potential etiologies for acute, spontaneous inflammatory conditions leading to unilateral blindness is lengthy, although predominantly infectious in nature. Polybius stated that the process was painful, but gave no indication of a systemic illness, suggesting that septic conditions, such as bacterial or fungal endophthalmitis, are unlikely to have been involved. No mention in the account is made of a rash or skin lesions, weighing against conditions such as smallpox or herpes zoster ophthalmicus.

Today, syphilitic keratitis would be considered as a differential diagnosis of this manifestation, but it can be excluded because the condition does not appear in Europe until far later than Hannibal’s march (*12*). A variety of other infections are well known to cause blindness, particularly in developing nations today. These infections include Whipple’s disease, leishmaniasis, leprosy, and onchocerciasis. However, they can be reasonably excluded in this instance because the chronic nature of these conditions is inconsistent with rapid onset described in this historical account.

## Pachydermal Zoonosis

Given Hannibal’s well-documented close contact with elephants (as mentioned, he was riding the sole remaining elephant at the time of his illness), zoonotic infections must be considered (*13*). One intriguing possibility would be leptospirosis. Elephants are known to frequently harbor the bacteria that causes this disease, which might be transmitted to humans through urine contact (*14*). However, leptospirosis rarely causes uveitis, and invariably causes bilateral disease when present, making this an unlikely cause.

Among possible alternatives, tuberculosis and brucellosis are both recognized to have potential for ocular involvement, including choroiditis and uveitis. Both *Mycobacterium tuberculosis* and *M. bovis* are known to infect elephants, and human-to-elephant transmission has been described (*15*). No systemic illness suggestive of disseminated tuberculosis was indicated by the historical account; however, ocular tuberculosis might rarely be manifested in isolation (*16*). Because tuberculosis was present in Europe during Hannibal’s era, this diagnosis cannot be excluded, although would be considered a particularly rare manifestation of disease and is probably less likely.

## Possible Solution

Perhaps the diagnosis most consistent with Polybius’ account is a form of keratitis. Keratitis might be caused by a range of pathogens, with or without preceding trauma, and is manifested typically with unilateral pain and visual disturbance (*17*). Untreated, it might result in permanent visual loss, as ultimately occurred in Hannibal’s case. The most common pathogens to cause keratitis are bacterial, including *Staphylococcus* spp. and *Streptococcus pneumoniae* (*18*). However, the timing of Hannibal’s illness arising in the context of a 3-day forced march through swamp and marshland, cannot be ignored (*19*). Although coincidental onset of an unrelated condition remains a possibility, the setting of illness strongly suggests a waterborne pathogen as the most likely etiologic agent. In particular, either *Pseudomonas aeruginosa* or *Acanthamoeba* keratitis would be consistent with the setting and clinical description of Hannibal’s illness.

Of the 2 forms of keratinitis, pseudomonal keratitis is more common and acute and perhaps the more likely. However, although *Acanthamoeba* keratitis in industrialized world settings today is most commonly associated with contact lens use, globally, it has been reported in the setting of either minor trauma or environmental exposure to contaminated water and so remains plausible as a cause of Hannibal’s condition given the environmental settings in which it originated (*20*,*21*). Seasonal variation in *Acanthamoeba* keratitis has also been reported from a variety of settings, although this appears to predominate in summer months and might in part be related to modern patterns of contact lens use (*21**,**22*).

In conclusion, although no definitive diagnosis can ever be established, we would argue that the historical record most strongly suggests keratitis caused by a waterborne pathogen, particularly *P. aeruginosa or Acanthamoeba*, as the cause of Hannibal’s ophthalmia. This diagnosis provides a more adequate account of his visual loss than traditional explanations have offered and, we hope, sheds a small additional light on the life of one of the greatest military leaders in world history.
